# Utilizing Nano-Adsorbents and Electrostatic Field Treatment for Sustainable Refinement of Crude Canola Oil

**DOI:** 10.3390/foods13172707

**Published:** 2024-08-27

**Authors:** Li Zhou, Timothy J. Tse, Farley Chicilo, Jianheng Shen, Venkatesh Meda, Martin J. T. Reaney

**Affiliations:** 1 Key Laboratory of Edible Oil Quality and Safety, State Administration for Market Regulation, School of Food Science and Engineering, Wuhan Polytechnic University, Wuhan 430023, China; zhou.li@usask.ca; 2 Department of Food and Bioproduct Sciences, University of Saskatchewan, Saskatoon, SK S7N 5A8, Canada; timothy.tse@usask.ca (T.J.T.); farley.chicilo@usask.ca (F.C.); jis956@mail.usask.ca (J.S.); 3 Department of Chemical and Biological Engineering, University of Saskatchewan, Saskatoon, SK S7N 5A9, Canada; 4 Prairie Tide Diversified Inc., Saskatoon, SK S7J 0R1, Canada; 5 Guangdong Saskatchewan Oilseed Joint Laboratory, Department of Food Science and Engineering, Jinan University, Guangzhou 510632, China

**Keywords:** canola oil, neutral oil loss, nano-adsorbent, electrostatic field, separation

## Abstract

Removal of polar impurities, such as phospholipids, free fatty acids (FFA), and peroxides, can be challenging during the refining of crude canola oil. Current conventional refining methods are energy-intensive (e.g., hot water washes) and can generate significant waste (e.g., wastewater effluent) and neutral oil loss. This study investigated the joint use of nano-adsorbents and electrostatic field (E-field) treatment as a potential and sustainable alternative in removing these impurities during the oil refining process. Specifically, aluminum oxide (Al_2_O_3_) nanoparticles were employed to neutralize FFAs, achieving a 62.4% reduction in acid value while preserving the fatty acid profile of the oil. After refining, E-field treatment was successful in removing the spent nano-adsorbent from solution (up to 72.3% by weight), demonstrating enhanced efficiency compared to conventional methods (e.g., gravitational settling, filtration, and centrifugation). The neutral oil loss using Al_2_O_3_ nano-adsorbents was also comparable to conventional refining methods, with a 4.38% (by weight) loss. After E-field treatment, the Al_2_O_3_ nano-adsorbent was then calcined to assess reusability. The Al_2_O_3_ nano-adsorbent was effectively recycled for three refining cycles. the methods do not use of large amounts of water and generate minimal waste byproducts (e.g., effluent). Nonetheless, while the nano-adsorbents demonstrated promising results in FFA removal, they were less effective in eliminating peroxides and pigments. E-field techniques were also effective in removing spent nano-adsorbent; although, optimization of E-field parameters could further improve its binding capacity. Finally, future studies could potentially focus on the physicochemical modifications of the nano-adsorbent material to enhance their refining capacity and reusability. Overall, this study presents a sustainable alternative or addition to conventional refining methods and lays the groundwork for future research.

## 1. Introduction

The global production of canola oil, produced from *B. napus* (L.) or *B. rapa* (L.), is expected to increase to 33.6 million metric tonnes by 2030 [1]. Canola oil is a rich source of health-promoting compounds, including triacylglycerols (TAG), micro-nutrients (e.g., carotenoids, tocopherols, and phytosterols) [2,3], and esters of essential fatty acids (e.g., linoleic acid and α-linolenic acid) [4]. Unfortunately, current conventional refining processes (e.g., deodorization) can contribute to a reduction in these nutritious compounds by up to 50% [2]. In addition, crude canola oil can contain impurities, such as partial glycerides (mono- and diglycerides), phospholipids, free fatty acids (FFAs), pigments, and peroxides [5,6]. The presence of these impurities can adversely affect edible oil properties of, including moisture content [7], phospholipid agglomeration [8], changes in oil appearance [9], and the production of pungent odors and flavors [10].

Many oil impurities are removed during refining. Conventional oil refining processes utilize water degumming methods to remove hydratable phospholipids [11], typically at elevated temperatures [12]. Alkaline treatments and water washing processes are then employed to neutralize FFAs (e.g., reduce oil acidity by converting FFAs into soap and water) [3]. The introduction of moisture during the neutralization process can degrade oil quality and impact bleaching performance during oil refining [13]. After neutralization, bleaching methods, using bleaching earth/clays or activated carbon, are applied to the oil to remove pigments (e.g., chlorophylls and carotenoids) [14] and oxidation products (e.g., peroxides). These processes typically occur under vacuum and in the presence of heat. Finally, the last step of the oil refining process is deodorization, where steam-stripping (e.g., steam distillation at elevated temperatures and under vacuum pressure) is applied to the oil to remove FFAs [15], volatile and oxidative compounds (e.g., aldehydes and ketones), and other impurities based on their vapor pressure and volatility [16]. The deodorization process helps to improve the flavor and oxidative stability of the refined oil. However, due to the harsh operating conditions, during deodorization, nutritional components (e.g., carotenoids, tocopherols, and phytosterols) in the oil are often prone to degradation [16]. Altogether, these conventional refining processes often require high temperatures, large energy inputs [17], and substantial water and chemical treatments. This further results in increased waste production [18], an increased potential for neutral oil loss [19], and degradation of nutritious compounds. Therefore, alternative sustainable refining strategies should be explored to remove these impurities while reducing environmental impacts and minimizing the degradation of nutritional compounds.

Recently, various adsorbents have been explored in oil refining processes to remove polar impurities, such as phospholipids, FFAs, pigments, and peroxides, from solution [20,21,22]. The performance of these adsorbents relies on their physiochemical properties (e.g., pore size, pore size distribution, and specific surface area) [23]. However, a notable drawback to adsorbents is their inherent ability to entrap oil triglyceride molecules within the pores on the absorbent surfaces and between adsorbent particles, which can lead to significant neutral oil loss [24]. More recently, nanoparticles (<100 nm) have been investigated in mitigating the loss of neutral oil due to their large surface area and abundance of active adsorption sites [25]. Nanoparticles have previously been used in refining aqueous solutions [26] and can offer a novel alternative in refining edible oils (e.g., vegetable oils) [27]. In addition, pre-treating nanoparticles with high temperatures and moisture (e.g., pre-hydrating) can improve their adsorption properties during oil refining [28] enhance the removal of polar impurities from non-polar triglycerides [29]. However, removal of nano-adsorbents can be significantly more challenging than their larger micro-adsorbent counterparts due to their size and physicochemical properties [30].

Electrostatic field (E-field) methods can remove both polar and oil-insoluble particulate materials (e.g., wear particles, soot, varnish, and dust) from dielectric oils (e.g., lubricating and hydraulic oils) [31]. For example, E-field technologies have been successful in removing carbon nanofibers [32] and other polar impurities from canola oil [33] and fatty acid methyl esters [22]. These technologies can rapidly remove particles and impurities from oil, based on their polarity or charge [34], making them an attractive alternative to conventional oil refining strategies. Adoption of both nano-adsorbent and E-field processes can offer an efficient and sustainable method in removing impurities from edible oil, thereby enhancing the refining process.

The purpose of this study was to investigate the combined applications of nano-adsorbent and E-field technologies in refining edible oils using crude canola oil as an example. This research examined three different nanoparticles (Al_2_O_3_, SiO_2_, and TiO_2_) for their capacity to remove common crude canola oil impurities. E-field treatment was then applied to assess its effectiveness in removing the spent nano-adsorbents from the oil. This study aimed to (1) examine the effects of moisture content and temperature on the adsorption capacity of the nano-adsorbents; (2) evaluate E-field efficiency in recovering the spent nano-adsorbents; (3) assess the reusability of spent nano-adsorbents; (4) investigate the impact of nano-adsorbent treatment on triglyceride structures; and (5) quantify the neutral oil loss incurred during the refining process. Successful applications of nano-adsorbent and E-field treatments could provide producers with a novel refining strategy for upgrading crude canola oil and other vegetable oils without the need for increased water and high-energy conventional systems.

## 2. Materials and Methods

Canola seed (*Brassica rapa* L.) was generously provided by Dr. Kevin Falk from Agriculture and Agri-Food Canada (Saskatoon, SK, Canada). The seeds were crushed using a laboratory single-screw oil press (Komet CA-59G3, IBG Monforts Oekotec GmbH & Co., Mönchengladbach, Germany) to produce freshly pressed crude canola oil. The oil was then filtered through glass wool to remove large particulates prior to refining.

All chemicals used were of analytical grade or higher. Hexane (≥98.5%) and methanol (≥99.9%) were obtained from Fisher Scientific (Ottawa, ON, Canada). Anhydrous ethanol was purchased from Greenfield Global Inc. (Brampton, ON, Canada). *N*, *N*-Dimethyl formamide (≥99.5%) was purchased from USB Corporation (Cleveland, OH, USA). Meanwhile, glass wool, deuterated chloroform (>97%), aluminum oxide (Al_2_O_3_; <50 nm nano-powder, 99.5% purity), silicon oxide (SiO_2_; 5–20 nm nano-powder, 99.5% purity), and titanium oxide (TiO_2_; 21 nm nano-powder, 99.5% purity) nanoparticles were purchased from Sigma-Aldrich (Oakville, ON, Canada).

### 2.1. Effects of Nanoparticles on Crude Canola Oil Acid Value, Pigment Content, Peroxide Value, and Fatty Acid Composition

The effects of Al_2_O_3_, SiO_2_, and TiO_2_ nanoparticles on reducing acid value, peroxide value, and pigment content (chlorophyll and carotenoids) were examined under three treatment conditions: (1) nanoparticles mixed with 100 g of crude canola oil at room temperature (herein 20 °C); (2) nanoparticles hydrated with water (20 wt. %) prior to mixing with 100 g of crude canola oil at room temperature; and (3) nanoparticles mixed with 100 g of crude canola oil under vacuum at 120 °C. The adsorbent dosage, optimized in a previous study, was 2.5 wt. % for each treatment group [22]. Mixing was performed on a VWR 600 series hotplate (Radnor, PA, USA) with a magnetic stirrer at 600 rpm for 60 min. After mixing, samples were incubated at room temperature for 3 min to allow the two fractions to separate. The upper layer was then collected and centrifuged at 3850× *g* for 5 min using a Beckman Coulter Allegra X-22R centrifuge (Kansas City, MO, USA). The supernatant was retained for further analysis of impurities, including free fatty acids (FFAs), carotenoids, chlorophylls, and peroxides.

Canola oil FFA content was expressed as acid value (AV) based on the equivalent percentage of oleic acid following the AOCS Official Method Cd 5a-40. Meanwhile, carotenoid and chlorophyll content in canola oil (mg/kg) was determined using an ultraviolet–visible spectrophotometer (UV-3100PC, VWR International, Radnor, PA, USA) following the British standards methods of analysis B.S-684-2.20, 1977 and AOCS official method Cc 13k-13, respectively. The peroxide value (PV) in the canola oil samples, expressed as milliequivalent per kg oil (meg/kg), was determined following the AOCS Official Method Cd 8b-90. Finally, the fatty acid composition in the canola oil samples was determined using ^1^H-NMR spectroscopy by transferring 200 µL of canola oil into a 5-mm NMR tube containing 600 µL of deuterated chloroform as the solvent. ^1^H-NMR spectra were recorded at 500 MHz (AMX 500, NMR Bruker, Mississauga, ON, Canada) with 16 scans per spectrum and analyzed using TopSpin v3.6.2 (Bruker BioSpin GmbH, Billerica, MA, USA).

The results from these tests suggested that, of the three nano-adsorbents investigated, the Al_2_O_3_ nano-adsorbent demonstrated optimal neutralization capabilities on crude canola oil (further discussed below). Therefore, the Al_2_O_3_ nano-adsorbent was selected for further investigation into its neutralization capabilities, neutral oil loss, E-field removal from solution, and reusability. To further optimize the neutralization capabilities of the Al_2_O_3_ nano-adsorbents, single-factor experiments were conducted to assess (1) adsorbent dosage (1–3 wt. %), (2) adsorption time (10–60 min), (3) agitation speed (100–800 rpm), and (4) adsorption temperature (20–120 °C) on improving neutralization on crude canola oil. Once identified, the optimal adsorbent dosage (2.5 wt. %) was used for all downstream experiments.

### 2.2. Removal of Al_2_O_3_ Nano-Adsorbent from Canola Oil

A commercial electrostatic “oil cleaner” (Kleentek DOC-R3, Parker Hannifin Corporation, Lancaster, NY, USA) was employed to remove the Al_2_O_3_ nano-adsorbents from solution. The operation parameters included a fixed flow rate of 0.9 L min^−1^ and a voltage of 10–12 kilovolts alternating current. This system comprised a reservoir containing the sample mixture of crude canola oil and nano-adsorbents that was circulated through the electrostatic field (E-field) system into a second reservoir. One complete circulation through the E-field represented a single “pass”. Samples were collected after 10 passes and submitted to the Saskatchewan Research Council Environmental Analytical Laboratories (Saskatoon, SK, Canada) to determine the Al_2_O_3_ nano-adsorbent content, inferred through the measurement of elemental Al, in the refined canola oil samples.

### 2.3. Canola Oil Loss

Canola oil loss during refining was determined by weighing the canola oil adsorbed to the Al_2_O_3_ nano-adsorbents as a percentage of the total amount of crude canola oil that was treated by the nano-adsorbents. Briefly, Al_2_O_3_ nano-adsorbent (2.5 wt. %) was thoroughly mixed with crude canola oil (50 mL) and centrifuged at 3850× *g* for 5 min. The top layer of canola oil was then decanted, and the tube wall was wiped with cotton moistened with *n*-hexanes to remove excess canola oil residue. Deuterated chloroform (5 mL) and the internal standard (*N*, *N*-dimethylformamide) were then added to the sample, and 200 mL aliquot was transferred to a 5-mm NMR tube prior to ^1^H-NMR analyses (described previously). Neutral oil loss was calculated using the following equation:

(1) $$Neutral\;oil\;loss\left(\%\right)=\frac{Mass\;canola\;oil\;adsorbed\;or\;entrained\;in\;adsorbent}{Total\;mass\;of\;canola\;oil\;being\;processed}\times 100$$

### 2.4. Reusability of Al_2_O_3_ Nano-Adsorbent for Crude Canola Oil Neutralization

E-field treatments were used to remove spent Al_2_O_3_ nano-adsorbents from solution, which could then be retrieved and investigated for reusability for crude canola oil neutralization by examining its recyclability and adsorption capacity for FFA. Briefly, crude canola oil (50 mL) and Al_2_O_3_ nano-adsorbents (2.5 wt. %) were thoroughly mixed and then centrifuged at 3850× *g* for 5 min. The supernatant was decanted, and the tube wall was wiped with cotton balls moistened with *n*-hexane to remove excess canola oil residue. The pellet was also briefly rinsed three times with 30 mL of *n*-hexane, ethanol, and distilled water. After rinsing, the pellet was calcined at 600 °C using a Thermolyne Benchtop Muffle Furnace (Type 12900, Thermo Scientific, Hillsboro, OR, USA) for 3 h. The dried Al_2_O_3_ nano-adsorbent was then re-applied to fresh crude canola oil to determine its adsorption capacity; this was continued over three recycling cycles to determine its reusability.

### 2.5. Characterization of Al_2_O_3_ Nano-Adsorbent Using Electron Microscopy

Finally, to understand how the morphology of the Al_2_O_3_ nano-adsorbent contributes to the removal of impurities in oil, scanning electron microscopy (SEM) was utilized. A field-emission scanning electron microscope (Hitachi, SU8010, Tokyo, Japan) was employed to observe the size, shape, and surface features of the Al_2_O_3_ nano-adsorbents. Samples were prepared for SEM observations following the method described by Zhou et al. [22].

### 2.6. Statistical Analysis

All analyses described above were performed in triplicate, and the data are presented as the mean ± standard deviation (SD). Statistical analyses were conducted using the Statistical Package for the Social Science (SPSS) version 25.0 (IBM Crop., Armonk, NY, USA). One-way ANOVA and post hoc Tukey HSD (honestly significant difference) tests were used to observe significance changes in AV, PV, carotenoid content, chlorophyll content, and fatty acid content. Significant differences were reported at the 95% confidence interval (*p* < 0.05), and the figures were constructed using Origin version 9.9 (OriginLab Corporation, Northampton, MA, USA).

## 3. Results and Discussion

### 3.1. Nanoparticles in Crude Canola Oil Refining

**Figure 1:**
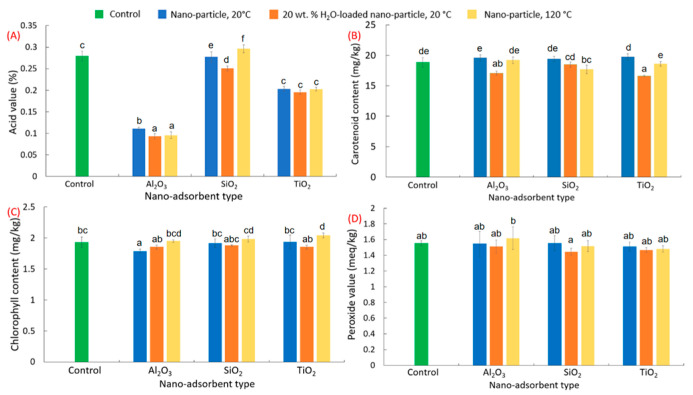
Screening of Al_2_O_3_, SiO_2_, and TiO_2_ nanoparticles (nanoparticles and hydrated nanoparticles at room temperature, and nanoparticles at 120 °C) on the removal of FFAs, carotenoids, chlorophylls, and peroxides from crude canola oil. (**A**) Acid value (FFA content), (**B**) carotenoid content, (**C**) chlorophyll content, (**D**) peroxide value. The letter above each bar plot denotes significance. All variables with the same letter indicate that the difference between the means was not statistically significant; meanwhile, variables with different letters indicate significance.

The refining capabilities of Al_2_O_3_, SiO_2_, and TiO_2_ nanoparticles on the removal of common impurities (FFAs, carotenoids, chlorophylls, and peroxides) from crude canola oil were investigated under different treatments (i.e., nanoparticle at room temperature, hydrated nanoparticle at room temperature, and nanoparticle at 120 °C under vacuum). Treatment with aluminum oxide (Al_2_O_3_) nanoparticles produced the greatest reductions in crude canola oil FFA content, or acid value, compared to the other nanoparticles investigated. Specifically, a reduction of 60.4% in acid value was observed in crude canola oil treated with dried Al_2_O_3_ nanoparticles at room temperature (Figure 1A). Comparatively, SiO_2_ and TiO_2_ nanoparticles produced oils with acid values that were decreased by 30.0% (Figure 1A). The performance of the Al_2_O_3_ nano-adsorbents were furthered improved by pre-hydrating them (20 wt. % with water) and increasing the adsorption temperature to 120 °C, resulting in an improved FFA removal of 6.2% (66.6% reduction) and 5.3% (65.6% reduction), respectively.

Although pre-hydrating the nano-adsorbent improves the acid value of canola oil, it can introduce moisture and lead to odors, which can further affect the quality, flavor, and shelf life of the refined oil product. Additionally, while higher temperatures (120 °C) can enhance the acid value, these methods often incur increased operational costs. Therefore, refining crude canola oil using Al_2_O_3_ nano-adsorbents at ambient conditions is preferable. Nonetheless, the Al_2_O_3_ nano-adsorbents demonstrated significantly better neutralization capacity compared to the SiO_2_ and TiO_2_ nano-adsorbents (one-way ANOVA, all *p*-values < 0.01). This superior performance could be attributed to the surface binding sites of Al_2_O_3_ and its capacity to bind to FFAs [35].

Meanwhile, pigment content (carotenoids and chlorophylls) and peroxide value were comparable among all nano-adsorbent and treatment groups and largely remained unchanged (Figure 1B–D) (one-way ANOVA, all *p*-values > 0.05). This indicates that these nano-adsorbents were ineffective in reducing some impurities as well as pigment content in edible oils, compared to conventional refining processes (e.g., bleaching). However, physicochemical modifications (e.g., surface modification) to nano-adsorbents can improve their binding capacity to adsorb and remove impurities [36,37], but this was not explored in this study. Future studies could investigate the physicochemical or morphological modifications of these nano-adsorbents in removing these compounds to improve oil flavor, odor, and nutritional quality. Nonetheless, as the Al_2_O_3_ nano-adsorbent demonstrated optimal performance in reducing FFA content compared to the other nanoparticles, it was selected for subsequent experiments described in this study.

Although not examined in this study, chemical modifications to nano-sized adsorbents have been previously investigated to enhance their adsorption properties [38]. For example, introducing functional groups (e.g., hydroxyl groups) has been demonstrated to improve the selective adsorption capacity of γ-Al_2_O_3_ adsorbents [39]. The chemical interaction between the hydroxyl groups on γ-Al_2_O_3_ adsorbents also increased binding affinity towards specific functional groups of the adsorbate (specifically those with carboxylate groups) [40]. Similarly, some micro-sized adsorbents that have been applied in crude edible oil neutralization have exhibited greater binding affinity to sodium hydroxide, resulting in reductions in free fatty acid content in the refined product [41]. This could describe the chemical interaction process between the Al_2_O_3_ nano-adsorbent and its ability in reducing free fatty acid content, as observed in this study, during canola oil refining.

### 3.2. Adsorptive Parameters of Al_2_O_3_ Nano-Adsorbent on Crude Canola Oil Acid Value

**Figure 2:**
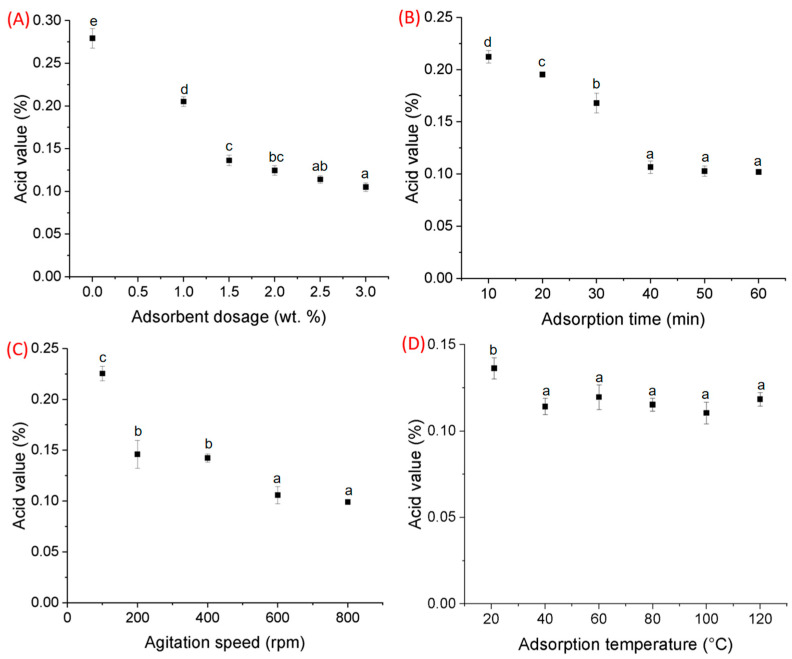
Adsorptive parameters of Al_2_O_3_ nano-adsorbent on crude canola oil neutralization. (**A**) Adsorbent dosage, (**B**) adsorption time, (**C**) agitation speed, and (**D**) adsorption temperature. The letter above each data plot denotes significance. All variables with the same letter indicate that the difference between the means was not statistically significant; meanwhile, variables with different letters indicate significance.

Significant reductions in canola oil AV were observed with increasing dosages of Al_2_O_3_ nano-adsorbent, up to 2.5 wt. % (one-way ANOVA test, *p*-value < 0.01). Specifically, AV reductions of 26.5% and 51.2% were observed in canola oil samples treated with 1.0 wt. % and 1.5 wt. % of Al_2_O_3_ nano-adsorbent, respectively (Figure 2A). Additionally, further reduction in AV from 51.2% to 62.4% was observed when the dosage was increased from 1.5 wt. % to 3.0 wt. % (Figure 2A). Consistent with previous research, a dosage of 2.5 wt. % Al_2_O_3_ nano-adsorbent was found to be optimal in reducing AV [22] in crude canola oil. This optimal dosage (2.5 wt. %) was used for all downstream experiments to determine optimal adsorption time, agitation speed, and adsorption temperature to improve crude canola oil neutralization.

Increasing the adsorption or contact time of Al_2_O_3_ nano-adsorbent during crude canola oil refining further improved the oil acid value (one-way ANOVA test, *p*-value < 0.01). Particularly, a reduction of 24.0%, 30.1%, 39.8%, and 61.9% in AV content was observed with 10, 20, 30, and 40 min of treatment, respectively (Figure 2B). Notably, extending the treatment time beyond 40 min resulted in minimal additional changes to the oil acid value. Similarly, increasing agitation speed resulted in significant reductions in crude canola oil AV when treated with Al_2_O_3_ nano-adsorbent (1.5 wt. %) at room temperature (21 °C) for 60 min (one-way ANOVA test, *p*-value < 0.01). Furthermore, although a reduction of 64.5% in crude canola oil AV was observed when agitation speed was increased to 800 rpm (Figure 2C), it was not significantly different from observations at 600 rpm (62.1% reduction). This suggests that agitation speeds > 600 rpm did not further improve oil acid value. Finally, an immediate and significant reduction of 51.2% in crude canola oil AV was observed when the operating temperature was increased to 40 °C (independent *t*-test, *p*-value < 0.05) (Figure 2D). Although a maximum reduction of 60.5% in AV was observed at 100 °C, significant differences in AV were not observed among different temperature treatments. Furthermore, increasing treatment temperature can also adversely effect canola oil micro-nutrients (e.g., carotenoids and tocopherols) [37,42] as well as require increased operational energy and capital requirements.

Similar to the findings described in Huang and Sathivel [43], increasing concentrations of Al_2_O_3_ nano-adsorbent were unable to completely remove FFAs from crude canola oil. In contrast, conventional alkaline treatment processes achieve total neutralization of oil [44]. Furthermore, the adsorption parameters used in this study, specifically a 40 min adsorption time at 600 rpm, were comparable to those employed in similar studies using adsorbents for vegetable oil neutralization [45]. In addition, increasing the reaction temperature did not enhance the adsorption efficiency of Al_2_O_3_ nano-adsorbent in crude canola oil neutralization. This could be attributed to the high energy requirements (up to 120 °C) for Al_2_O_3_ nano-adsorbents [46].

### 3.3. Al_2_O_3_ Nano-Adsorbent Removal from Canola Oil

**Figure 3:**
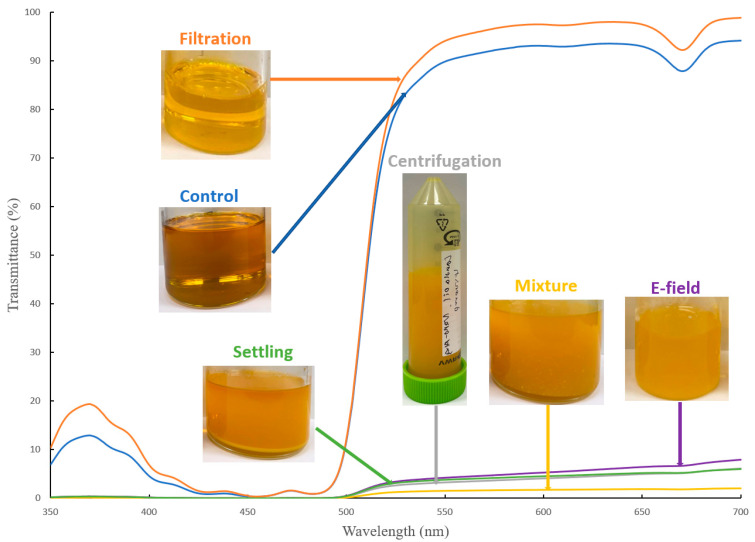
Transmittance of conventional methods (e.g., gravitational settling, centrifugation, filtration) and E-field techniques in removing nano-adsorbent from crude canola oil.

Although Al_2_O_3_ nano-adsorbents were successful in neutralizing crude canola oil, removal of these particles from crude canola oil can be challenging compared to larger-sized adsorbents. Conventional methods (e.g., gravitational settling and centrifugation) are ineffective in precipitating spent Al_2_O_3_ nano-adsorbents from crude canola oil (Figure 3) [22]. In addition, membrane filtration methods can result in blockages [22], making them impractical at an industrial scale. Therefore, alternative methods, such as E-field technologies, have been investigated, as they can rapidly separate impurities from solution and are more cost-effective compared to traditional separation methods (e.g., filtration) [47]. These techniques have demonstrated success in removing >70 wt. % of spent nano-adsorbents [22]. Similarly, in this study, efficient removal of spent Al_2_O_3_ nano-adsorbents also enabled removal rates of >72.3 wt. % of spent Al_2_O_3_ nano-adsorbents. The remaining Al_2_O_3_ nano-adsorbents present in the canola oil could be due to a lack of optimization of the E-field voltage, flow rate, and temperature applied during the refining process. In addition, the amount of canola oil that was mixed with nano-adsorbents may have exceeded the load capacity of the collector in the E-field system. For example, in a previous study, the removal rate of spent Al_2_O_3_ nano-adsorbent from fatty acid methyl esters (FAME) achieved a similar result of 79.3% [22], although this could be due to the lower viscosity of FAME.

Regardless, E-field techniques had demonstrated efficiency in removing nano-size adsorbents from high-viscosity fluids (e.g., canola oil) compared to conventional methods (e.g., gravitational settling, centrifugation, and filtration). These traditional methods are costly in terms of equipment, labor, and energy requirements while also demonstrating poorer efficiency [48]. Filtration methods are also impractical for high-viscosity solutions, as high pressures are required to improve flow rates [49], and frequent filter replacements are required due to blockage [50]. Meanwhile, E-field technologies offer operational advantages, such as the interaction of the nano-adsorbent against the E-field force can enhance its binding affinity to the collector while also minimizing blockages. Furthermore, the operational costs for these technologies are only affected by the electrical costs and the replacement costs for the collector (which is produced from folded cellulose-based paper). E-field efficiency can potentially be improved through the addition of chelating agents that can manipulate the physicochemical properties of the oil and nano-adsorbents and enhance the removal of impurities [51]. Nonetheless, this novel method offers a promising alternative in the removal of nanoparticles used during the oil refining process compared to conventional techniques [48].

### 3.4. Neutral Oil Loss

**Figure 4:**
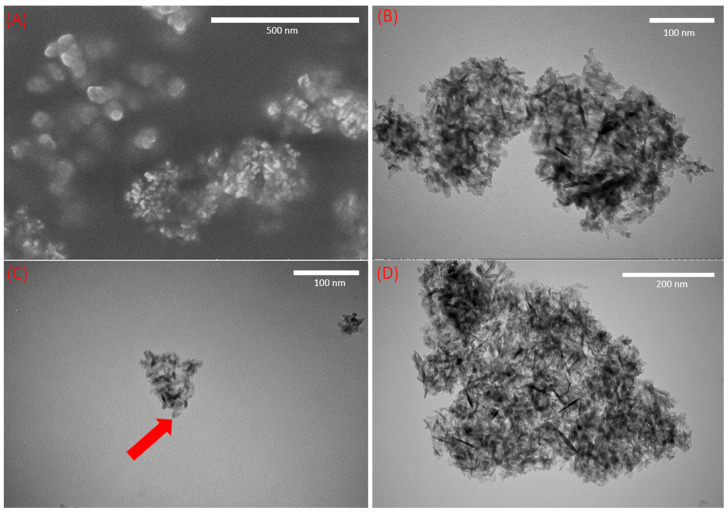
(**A**) SEM image of Al_2_O_3_ nano-adsorbents (100,000× magnification); (**B**) TEM image of un-treated Al_2_O_3_ nano-adsorbents (200,000× magnification); (**C**) TEM image of Al_2_O_3_ nano-adsorbent (200,000× magnification)—the consistency of each particle (highlighted by the red arrow) is uniform, indicating a homogenous texture and absence of cavities; (**D**) TEM image of Al_2_O_3_ nano-adsorbents contacted with canola oil and *n*-hexane washing (100,000× magnification).

The calculated canola oil loss was determined to be 4.38 wt. % (Equation (1)); however, this loss could be attributed to the triglyceride content, possibly entrained in the aggregated clusters of Al_2_O_3_ nano-adsorbent (Figure 4A,B). In addition, triglyceride molecules may also be bound to active sites of the Al_2_O_3_ nano-adsorbents, which can affect adsorption efficiency, decrease crude canola oil neutralization, and increase oil loss. Comparatively, conventional vegetable oil refining losses typically fall within the range of 2–5% [52].

### 3.5. Reusability of Al_2_O_3_ Nano-Adsorbent on Crude Canola Oil Neutralization

**Figure 5:**
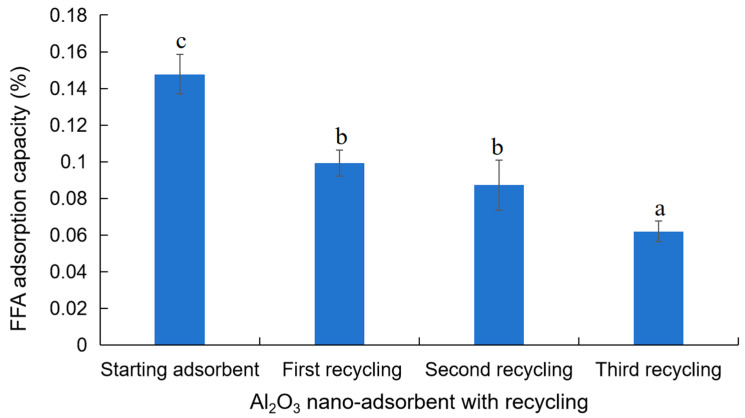
Reusability of Al_2_O_3_ nano-adsorbent in crude canola oil neutralization. The letter above each bar plot denotes significance. All variables with the same letter indicate that the difference between the means was not statistically significant; meanwhile, variables with different letters indicate significance.

Demonstrating reusability of nano-adsorbent materials is beneficial in promoting sustainability and reducing operational costs. In this study, a gradual decrease in Al_2_O_3_ nano-adsorbent efficiency was observed after each use. A decline of 32.8% in adsorbent efficiency was observed after the initial recycling of Al_2_O_3_ nano-adsorbent, inferred from its capabilities in reducing acid value (e.g., FFA removal). Additional recycling of Al_2_O_3_ nano-adsorbent resulted in a further decline in adsorption efficiency by 40.9% and 58.0% after the second and third recycle, respectively (Figure 5). To address this decline, solvent washing, using *n*-hexane, ethanol, and distilled water, was employed to remove non-polar and polar impurities prior to reactivating the nano-adsorbent via calcination. The decline in FFA removal efficiency after recycling could also be attributed to the presence of trace metals in the crude canola oil that can alter the adsorptive properties [42] or block the active sites of the Al_2_O_3_ nano-adsorbents [53]. In addition, calcination at extreme temperatures (>600 °C) can potentially change the morphological structure of the nano-adsorbent, further affecting its physicochemical properties [54].

Although calcination treatments can be restrictive in an industrial setting, due to its high energy requirements, the use of nano-adsorbents is still being investigated in its efficient capabilities in removing unwanted contaminants and impurities from solution. In this study, our research demonstrates that nano-adsorbents appear to be an effective alternative compared to conventional absorbents, based on their binding capacity in removing certain impurities. Our research also highlights the current limitations in implementing these strategies. Although not expanded on in this study, fabrication and modification of nano-adsorbents (e.g., surface modification methods) [36] might also significantly improve their physicochemical properties [55] and, their application in oil refining strategies. For example, the modification of clay nano-adsorbents through acid treatment and calcination greatly enhanced their capacity to absorb dyes from aqueous solutions [56]. Similarly, surface modifications of carbon-based nano-adsorbents improved their ability to decontaminate wastewater [57].

### 3.6. Canola Oil Fatty Acid Profile with Al_2_O_3_ Nano-Adsorbent Treatment

The predominant fatty acids (content > 2%) identified in the canola oil samples include palmitic acid (16:0), stearic acid (18:0), oleic acid (18:1), linoleic acid (18:2), and linolenic acid (18:3) (Table 1). Additionally, arachidic acid (20:0) and gondoic acid (20:1) were observed but in lower quantities (content < 2%) (Table 1). During the refining process using Al_2_O_3_ nano-adsorbents and E-field treatments, the fatty acid profile in the canola oil was not affected (one-way ANOVA test, all *p*-values > 0.05) (Table 1). Comparatively, conventional refining processes that require high temperatures or permit exposure to air can result in the hydrolysis and decomposition of the fatty acids in vegetable oils [9]. This study demonstrated that refining edible oils (e.g., crude canola oil) using Al_2_O_3_ nano-adsorbents can partially neutralize the oil without affecting its triglyceride structure. However, it is essential to consider the appropriate type of nanoparticle for oil refining processes, as certain materials, such as solid acid Al_2_O_3_ and base MgO, have been reported to decompose certain edible oils (e.g., soybean oil) [58].

### 3.7. Characterization of Al_2_O_3_ Nano-Adsorbent by Electron Microscopy

The morphology of the Al_2_O_3_ nano-adsorbents was examined via electron microscopy and demonstrated a tendency to aggregate and form clusters (Figure 4A,B) despite appearing to be cylindrical in shape with a smooth surface and lacking ‘cavities’ (Figure 4B,C). The presence of ‘cavities’ can increase neutral oil loss due to oil entrainment [59]. Similarly, neutral oil loss can also occur through the entrainment of compounds in the triglyceride molecules via nanoparticle aggregation. Notably, no obvious differences were observed between Al_2_O_3_ nano-adsorbents before and after contact with crude canola oil (Figure 4B,C). However, this lack of distinction could be attributed to the removal of canola oil impurities during *n*-hexane washes when samples were prepared for electron microscopy observations.

## 4. Conclusions

In summary, this study investigated the application of nano-adsorbent and E-field technologies for the neutralization of crude canola oil. A variety of nanoparticles (Al_2_O_3_, SiO_2_, and TiO_2_) were evaluated for their adsorption efficiency in refining crude canola oil. In addition, E-field treatments were applied as a novel method for the removal of spent nano-adsorbents. Aluminum oxide (Al_2_O_3_) nano-adsorbents demonstrated superior neutralization effects on FFAs in crude canola oil, achieving a reduction of 62.4% in acid value. This performance was significantly more efficient compared to SiO_2_ and TiO_2_ nanoparticles. While nano-adsorbents effectively removed FFAs, they did not significantly reduce peroxide or pigment contents, highlighting the specificity of nano-adsorbents towards certain impurities and underscoring the need for a multifaceted approach in edible oil refining or the application of physicochemical modifications to improve their refining capabilities.

The implementation of E-field treatments after nano-adsorbent applications offers a unique alternative for separating spent Al_2_O_3_ nano-adsorbents from the refined oil, successfully removing 72.3 wt. % of the adsorbents. This represents a breakthrough in overcoming the challenges associated with recovering spent nano-adsorbents from solution. These approaches achieved comparable neutral oil loss (typically between 2 and 5%) with conventional refining processes, recording a neutral oil loss of 4.38 wt. % when using Al_2_O_3_ nano-adsorbents. Furthermore, examination of the reusability of Al_2_O_3_ nano-adsorbents revealed a decrease in adsorption efficiency over three cycles, suggesting a potential area for further optimization or reactivation strategies.

Comparatively, the conventional method for crude edible oil neutralization involves the use of an alkaline solution like sodium hydroxide, which reacts with free fatty acids to form soap which is then removed with hot water washes. This process, which includes multiple steps such as centrifugation and drying, can incur varying operational costs based on plant and production line scale as well as environmental requirements (e.g., reclamation of wastewater effluent). Comparatively, micro-sized adsorbents have also been explored in reducing free fatty acid content. The use of these adsorbents can potentially minimize the costs associated with hot water washing and wastewater treatment. While the removal of micro-sized adsorbents using techniques like gravitational settling, centrifugation, and filtration has demonstrated success, these methods can also be time-consuming and require frequent filter replacements.

Meanwhile, nano-adsorbents (specifically Al_2_O_3_ nano-adsorbents) coupled with E-field technologies were successful in the removal of both common impurities, such as free fatty acids, and the subsequent elimination of spent nano-adsorbents without the requirement for chemical additives or significant wastewater production. Moreover, E-field methods rapidly removed spent nano-adsorbents compared to conventional methods, suggesting an increase in process refining flow rates and end-product volumes. While recognizing the limitations in the reusability of nano-adsorbents, this study highlights the promise of future research into the physicochemical properties of chemically or surface-modified adsorbents. These advancements could potentially enhance adsorption binding capacity and reusability, thus improving the performance of adsorbent materials in edible oil refining.

In conclusion, the integration of nano-adsorbent and E-field technologies in edible oil refining offers an alternative and sustainable approach that is less resource-intensive and potentially more environmentally friendly compared to conventional methods. These methods demonstrated promise in maintaining the fatty acid profile in the refined canola oil while removing FFAs. In addition, the recyclability of the nano-adsorbent was limited, which can provide areas for future research and development. Altogether, this study paves the way to further develop innovative methods that can substantially benefit the edible oil industry by enhancing the quality of oil and reducing the environmental footprint of the refining process.

## Figures and Tables

**Table 1:** Fatty acid composition of canola oil samples with and without Al_2_O_3_ nano-adsorbent treatment.

Fatty Acid Type	Untreated Canola Oil (%)	Al_2_O_3_ Nano-Adsorbent Treated Canola Oil (%)
C16:0	5.01 ± 0.03	5.00 ± 0.05
C18:0	2.50 ± 0.02	2.50 ± 0.02
C18:1	66.52 ± 0.07	66.57 ± 0.06
C18:2	21.73 ± 0.04	21.70 ± 0.03
C18:3	2.43 ± 0.02	2.43 ± 0.05
C20:0	0.75 ± 0.01	0.75 ± 0.01
C20:1	1.06 ± 0.01	1.05 ± 0.01

## Data Availability

The original contributions presented in the study are included in the article, further inquiries can be directed to the corresponding authors.
